# Building a Layer-Structured Aluminum/Graphene Composite with Significant Improvement in Electrical Conductivity

**DOI:** 10.3390/ma17122979

**Published:** 2024-06-18

**Authors:** Zishun Wang, Wenhui Li, Junliang Ma, Baishan Liu, Wei Wang, Zhaoping Liu

**Affiliations:** 1 School of Material Science and Chemical Engineering, Ningbo University, Ningbo 315211, China; wangzishun@nimte.ac.cn (Z.W.); 13755934653@163.com (W.L.); 2 Key Laboratory of Graphene Technologies and Applications of Zhejiang Province, CAS Engineering Laboratory for Graphene, Ningbo Institute of Materials Technology & Engineering, Chinese Academy of Sciences, Ningbo 315201, China; majunliang@nimte.ac.cn; 3 CRRC Industrial Academy Co., Ltd., Beijing 100039, China; liubaishan@crrcgc.cc

**Keywords:** graphene, Al, composite, electrical conductivity

## Abstract

Aluminum (Al) and its alloys are widely used in various fields due to their excellent physical properties. Although many efforts have been made to fabricate an Al-based composite, they usually results in a significant decrease in electrical conductivity. Herein, a special layer-structured Al/graphene (Gr)/Al composite was successfully designed and fabricated through a facile method using the ultrasonic spraying of graphene powder with alumina removal and a subsequent vacuum hot-pressing process. The as-obtained Al/Gr/Al composite presents a significantly enhanced electrical conductivity of 66% IACS, which is much higher than that of other reported Al-based composites, while it still maintains similar mechanical properties. This work provides a new strategy for the development of highly conductive Al-based composites, which would be very useful and important for practical applications.

## 1. Introduction

Aluminum (Al)-based composite is one of the most widely used metal composite materials due to its excellent physical properties of high specific strength, low density, high impact resistance, low thermal expansion coefficient, high specific modulus and good wear resistance [1,2,3]. There is a sustained demand to improve the electrical and mechanical performance of Al-based composite toward the dramatically increased development of new energy fields [4,5]. However, up to now, many works have been created to improve mechanical strength of Al-based materials, while few works have reported higher electrical property [6].

Alloying is one of the most commonly used manufacturing methods to improve the strength of aluminum, but it usually results in a decrease in electrical performance [7]. For example, Abbas et al. designed a high content of Al-5Zr (wt%) alloy through large plastic deformation with the alloy microhardness up to 150 Hv, but the increase in Zr element solid solution led to a significant decrease in the electrical conductivity of the alloy to 35% IACS [8]. By introducing rare earth elements, the microstructure of the alloy can be refined, and solute elements can be precipitated as a second phase, which can improve the electrical properties of the alloy to some extent [9]. Wang et al. developed an Al-Y alloy with a eutectic system, and the alloy strength improved to 232 MPa, while the eutectic structure led to a significant decrease in the corresponding electrical conductivity to 52.24% IACS [10]. On the other hand, process optimization is a traditional method to improve the performance of aluminum alloys, which aims to improve the internal organization of the alloy and reduce the influence of elements with high solubility on the electrical conductivity [11]. Zhou et al. processed Al-0.2Sc-0.04Zr (wt%) via cold rolling and aging treatment to form fine Al_3_(ScZr) precipitates with improved intergranular structure, which present the best tensile strength of 213 MPa and a lower electrical conductivity of 61.7% IACS [12]. However, it is known that the solid solution of alloy elements will cause lattice distortion in the aluminum matrix and form the Guinier Preston zone [13,14,15], which becomes the scattering source of electron movement [16,17] and leads to a significant decrease in the electrical conductivity of the aluminum alloy. Thus, selecting a suitable reinforcing phase with excellent electrical performance is still the bottleneck for aluminum composites.

Graphene (Gr) is a new carbon material used for many applications and presents high strength, a high elastic modulus, high electrical conductivity and excellent thermal conductivity [18,19,20,21,22]. It is considered an excellent, promising reinforcement to prepare graphene–aluminum-based alloy composites with improved physical properties [23]. However, current research on graphene–aluminum composites mainly focuses on improving their mechanical properties, while there are only a few reports on enhancing electrical conductivity. One should note that graphene cannot grow in situ on an aluminum metal matrix, and aluminum is naturally oxidized in the air to form Al_2_O_3_, which can hinder the molding of aluminum-based composites. Therefore, current research mainly mixes graphene powder and aluminum metal powder for preparing Al/Gr composites materials using methods including powder metallurgy [24], liquid casting [25] and 3D printing [26]. Because of the strong van der Waals forces between the graphene layers and the high surface area, the agglomeration usually occurs in the graphene powders, which needs to be overcome for a better performance. Yu et al. prepared Gr/Al6063 composites using mechanical milling and the pressure infiltration method. With the uniform distribution of graphene achieved by 3 h of ball milling, the maximum tensile strength of the composite reached 276 MPa, and the conductivity of the composite was only 52.8% IACS [24]. The long ball-milling time caused an increase in defects in the graphene, and hence Al_4_C_3_ grew in large quantities at the graphene defects, which hindered the electron transfer. Kim et al. dispersed liquid graphene oxide (GO) on the surface of aluminum powder and prepared aluminum/reduced graphene oxide (rGO) composites by thermal reduction. The composite containing 0.2 vol% rGO showed a yield strength of 374 MPa [27]. However, the presence and defects of oxygen functional groups in GO and rGO reduced the conductivity to only 51% IACS. Chyada et al. treated Al/Gr composites prepared by the liquid casting method with an aging treatment, and the conductivity of the composite increased to 63.27% IACS, slightly higher than that of pure aluminum [28]. Recent studies have shown that a layered composite of copper and graphene can combine the high electron mobility of graphene and the high electron density of copper, improving the carrier mobility and carrier density at the same time, with a breakthrough of achieving 117.4% IACS [29]. At the same time, for the same layered structure, the current of the Gr in the A1/Gr/Al interface structure is 40 times higher than that of the Al on both sides [30], which brings new possibilities for the aluminum–graphene composite.

In this work, a layer-structured Al/Gr composite was designed and fabricated by hot pressing graphene-coated Al foil with oxide removal. The good interconnection between Al layers with the graphene layer at the interface enabled a higher electrical conductivity with a slightly higher strength as well, which provides a new method for the preparation of highly conductive Al/Gr composites for practical applications.

## 2. Materials and Methods

### 2.1. Materials

PG (5 wt% viscous aqueous slurry), which is prepared by the interaction and exfoliation of graphite and has a mean thickness of 2.4 nm (approximately 7 layers), was supplied by Ningbo Morsh Technology Co., Ltd., Ningbo, China. Rolled aluminum foil at a purity of 99.95% and thickness of 20 μm was provided by Hangzhou Five Star Co., Ltd., Hangzhou, China. Chromium trioxide (CrO_3_) at a purity of 99.99% was supplied by Shanghai Aladdin Biochemical Technology Co., Ltd., Shanghai, China.

### 2.2. Fabrication of Al/Gr Composite Material

First, a Gr (2 wt% viscosity water slurry) dispersion was obtained by diluting the PG (5 wt% viscosity water slurry) with DI water using an abrasive disperser. Subsequently, the slurry was treated by an ultrasonic process at 300 W power for 60 min to obtain a gray dispersion. Then, the gray dispersion was loaded into a UC360C ultrasonic precision spraying machine to spray Gr onto the aluminum substrate, and the thickness of the Gr film was adjusted by controlling the number of the spraying loop and the speed of the loading liquid. After spraying, the as-obtained Al/Gr composite foil was cut into a square of 20 × 20 mm in size by using a laser cutting machine. To remove the oxide layer on the aluminum foil surface, the resulting Gr/Al sheet was immersed in a mixed acid solution, which was prepared by dissolving H_3_PO_4_ (25.532 g) and CrO_3_ (6.124 g) in 400 g deionized water at 80 °C for 8 h, and was finally washed with isopropanol. The as-obtained Al/Gr composite samples were quickly laminated and put into a hot press and then heated to the specified temperature at 10 °C/minute under a vacuum. Then, each sample was kept at a designated pressure at this temperature for 1 h. Finally, the sample was obtained by cooling the furnace to 200 °C at 10 °C/min and then to room temperature naturally.

### 2.3. Characterization of Al/Gr Composite

Raman spectroscopy (Renishaw inVia Reflex) was performed by using a 532 nm laser as the excitation source to characterize the quality and layers of graphene in the Al/Gr composite foils. The morphology and distribution of graphene on the surface of the Al foil and the microstructures of the Al/Gr laminar composite were characterized using a scanning electron microscope (SEM, S-4800) and an optical microscope (OM, L-3800).

X-ray diffraction (XRD) analysis of the composite was performed on a Bruker D8 ADVANCE X-ray diffractometer using Al K α radiation. The diffraction range was 20°~100°, with a scanning rate of 4°·min^−1^. The electrical conductivity of the prepared Al/Gr composite was determined using the van der Pauw method, and all the samples were wire cut into a dimension of 20 mm × 20 mm × ~0.6 mm. The thickness of the Al/Gr bulk composite was calculated by measuring density, weight and length/width.

## 3. Results and Discussion

**Figure 1:**
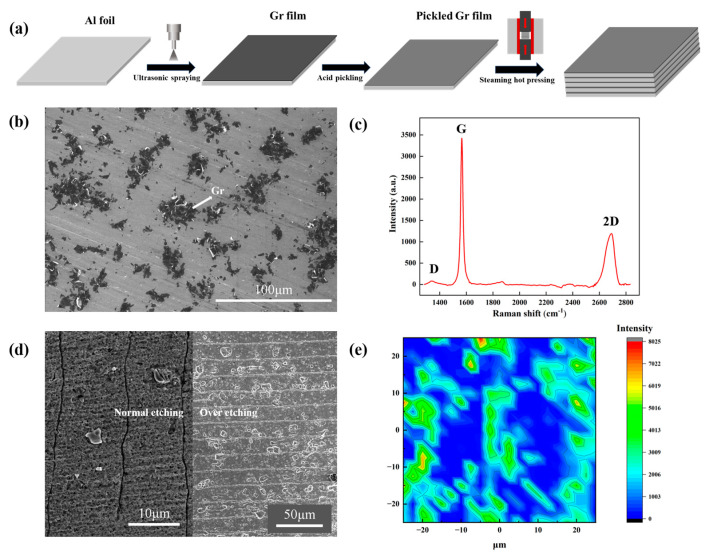
(**a**) Scheme of the fabrication process for layer-structured Al/Gr composites. (**b**) SEM image of the surface morphology of the graphene-coated Al foil. (**c**) Raman spectra of the graphene powder sprayed on the Si substrate. (**d**) SEM images of the cross section of Al/Gr layered composites. (**e**) Raman spectra of the graphene-coated Al foil after acid treatment.

The typical fabrication process of Al/Gr composites is shown in Figure 1a. With the graphene deposited on one side of pure aluminum foil as a building block, the layer-structured Al/Gr composite can be obtained by stacking and hot pressing several layers of building blocks. It is worth mentioning that the soaking process in mixed acid plays the key role in removing the native oxide on the Al foil and hence enabling excellent bonding between layers after the hot-press process. In comparison, obvious stratification phenomena can be observed in the Al/Gr composite without soaking in mixed acid, as shown in Figure S1 in the supporting information. Figure 1b presents the surface morphology of the graphene-coated Al foil. The corresponding Raman spectra in Figure 1c indicates the nature of the few-layer graphene powder, where the relative intensity (ID/IG) between peak D and peak G is about 0.04. After the acid treatment to remove the native Al_2_O_3_ on the surface, the coverage of graphene on the Al surface becomes lower during the washing process [31]. Figure 1d presents cross-sectional SEM images of the Al/Gr composite. Due to the good ductility of Al, it is quite hard to distinguish the interface in the layer-structured Al/Gr composite, as shown in the left part of Figure 1d. Over-etching in acid is additionally performed, which clearly illustrates the layer structure of the Al/Gr composite, as shown in the right part of Figure 1d. The Raman mapping in Figure 1e demonstrates the uniform distribution of graphene on the Al surface, which is favored for the formation of a uniform Al/Gr composite in the following hot-press process.

**Figure 2:**
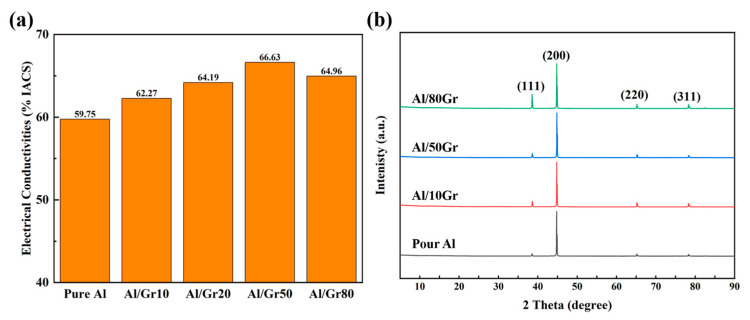
(**a**) The electrical conductivities of Al/Gr composites with different graphene spraying times. (**b**) XRD patterns of Al, Al/Gr10, Al/Gr50 and Al/Gr80 composites after hot pressing at 655 °C with a pressure of 50 MPa.

Figure 2a presents the electrical conductivities of the Al/Gr composites with different graphene contents. The original Al foil exhibits an electrical conductivity of 59–60% IACS, which is very close to that of an ideal Al material, and the slight difference might come from the contacting resistance of alumina and measurement error. After introducing Gr powder into the composite, the corresponding electrical conductivity increases with the increasing content of graphene, and the highest value reaches 66.63% IACS for the Al/Gr50 sample after spraying the graphene solution on the Al foil 50 times as building blocks. Compared with the conductivity of pure aluminum, there is a significant increase of about 11%. However, after further increasing the content of graphene by increasing the spraying cycles to 80 times, the electrical conductivity of the corresponding sample starts to decrease. This might be because the increase in sprayed graphene would agglomerate seriously on the surface of the Al foil and hence affect the interface connection of the composite material. The XRD patterns of pure Al and Al/Gr composites are shown in Figure 2b, which presents the diffraction peaks of Al since the graphene content is relatively low. Related studies have shown that Al and graphene react at high temperatures to form Al_4_C_3_, which nucleates and grows in the graphene defects and thus affects the physical properties [32,33,34]. In this work, by spraying graphene powder, the damage to graphene in the ball-milling process would be avoided, and the formation of Al_4_C_3_ could be greatly reduced. Through the XRD analysis of the material, it can be proven that the Al grain orientation of the Al/Gr layered composites has not changed significantly, and the formation of Al_4_C_3_ is negligibly observed.

**Figure 3:**
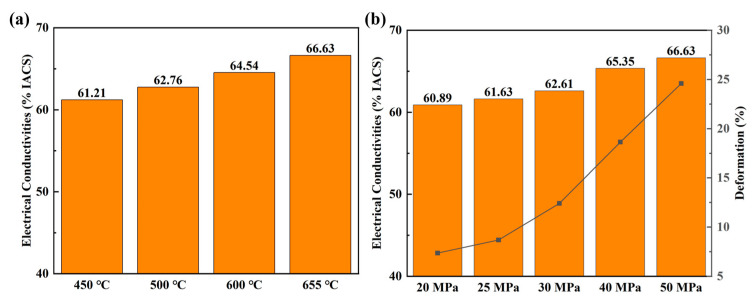
(**a**) The electrical conductivities of Al/Gr50 composites with hot pressing at different temperatures and at the same pressure of 50 MPa, and (**b**) the electrical conductivities of Al/Gr50 composites with hot pressing at different pressures and at the same temperature of 655 °C.

In order to further understand the phenomena of enhancing electrical conductivity in the Al/Gr layered composites, the electrical performance of composites fabricated at different processing conditions was studied in detail. Figure 3 presents the electrical performance of Al/Gr50 bulk composite samples fabricated at different temperatures and pressures. As shown in Figure 3a, with an increasing hot-pressing temperature, the electrical conductivity of the Al/Gr50 composite gradually increases from 61.63% IACS for hot pressing at 450 °C to 66.63% IACS for that at 655 °C. On the other hand, with an increasing hot-pressing pressure, the electrical conductivity of Al/Gr50 composite also increases from 60.89% IACS for pressing at 20 MPa to 66.63% IACS for that at 50 MPa. The result indicates that a higher hot-pressing temperature and higher hot-pressing pressure is better for achieving higher electrical performance of the Al/Gr composite. In the meantime, the deformation of Al/Gr is also carefully examined, which is well known to have a similar trend with the change in hot-pressing temperature and pressure. As demonstrated in Figure 3a,b, the deformation ratio increased significantly with increasing the hot-pressing temperature and pressure, which suggests that the increase in electrical conductivity of the Al/Gr composite is related to the change in composite structure. As a comparison, in the layered Cu/Gr/Cu configuration, with a decreasing distance of the Cu–Gr interface, the contribution of copper atoms and carbon atoms near the Fermi level increases, and graphene forms a bridge with electron conduction at a small Cu–Gr distance for enhancing the electrical conductivity [35].

**Figure 4:**
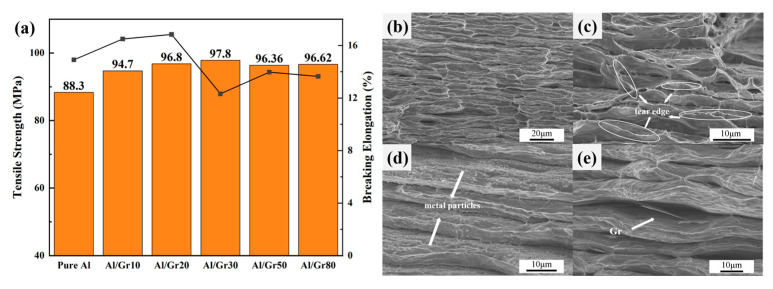
(**a**) Engineering stress–strain curves for Al/Gr composites and the unreinforced Al matrix fabricated by hot pressing at 50 MPa and 655 °C; the bar chart represents tensile strength, and the line chart represents fracture strain. Cross-sectional SEM images of (**b**) pure Al, (**c**) Al/Gr10, (**d**) Al/Gr30 and (**e**) Al/Gr50 composites.

Based on the above discussion, the change in micro-structure presents a similar relationship with the electrical performance of the Al/Gr composite, which would also affect the mechanical properties. Herein, tensile tests are performed to evaluate the strength and elongation of as-prepared Al/Gr composites. Figure 4a shows the representative engineering stress–strain curves of Al/Gr bulk composites with different spraying cycles, which are all hot pressed based on 655 °C and 50 MPa, as well as those of the non-reinforced Al matrix prepared under the same process conditions without graphene coating. It can be seen that the tensile strength of all Al/Gr composites is improved compared that of the pure Al matrix. The highest value reaches 97.8 MPa in the Al/Gr30 composite, which is about 11% higher than that of the Al matrix. The improvement is not very significant compared to that of previous reports since the graphene content in this layer-structured Al/Gr is quite small. However, the engineering strain is not changed obviously. The engineering strain of only Al/Gr10 and Al/Gr20 is slightly higher than that of the pure Al matrix, which indicates that the introduction of more graphene would decrease the engineering strain of the Al/Gr composite. This result is consistent with other reports that, due to the difference in hardness and tensile properties between graphene and aluminum substrate, the hard lamellar structure of graphene itself increases the brittleness of the material while increasing the yield strength [36,37].

The fracture morphologies of Al/Gr composites and pure aluminum are studied in detail. In Figure 4b–e, it can be seen that there is a layering phenomenon that exists in both the pure aluminum samples and the Al/Gr composites. Under the action of tensile load, the stress concentration occurs in the inter-layer defects, and the failure occurs first, which might explain why the tensile performance of as-prepared composites is not very high. As shown in Figure 4b, the pure aluminum sample presents a tear edge at the fracture. However, for the Al/Gr composites with a layer structure as shown in Figure 4c–e, the appearance of the dimple is not typical, but the irregular tear edge can still be interpreted as a ductile fracture. For example, through the cross-sectional image of the Al/Gr30 sample in Figure 4d, it can be found that there are fine metal particles between the layers, which might be due to the aluminum metal particles etched in the pickling process. These aluminum metal particles exist in the layers of the composite material and play a pinning role in the process of tensile deformation to prevent further deformation. Therefore, the tensile strength of Al/Gr30 composites is increased, while the ductility of the material is reduced. As shown in Figure 4e, edge fractures in the graphene can be observed between layers, which indicates that parts of the tensile load will be transferred from the aluminum matrix to the graphene through interfacial shear stress during stretching and hence lead to the graphene fracture. The high strength of graphene directly improves the mechanical strength of the composite material, which is consistent with the reported strengthening mechanisms of Al/Gr composite materials [38,39].

**Figure 5:**
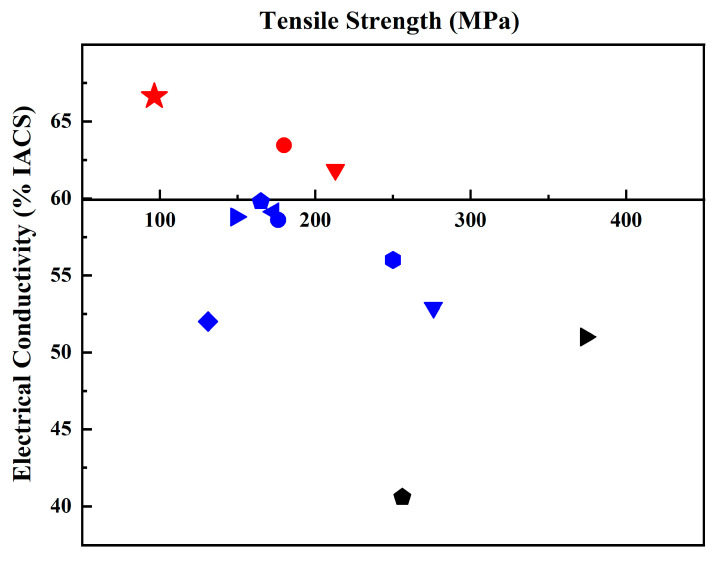
Comparison of tensile strength and electrical conductivity of the Al/Gr composites in reported Al/Gr composites. The theoretical electrical conductivity and tensile strength of pure aluminum are taken as the coordinate origin. (The detailed information of all Al/Gr composites in the figure is listed in Table 1).

Figure 5 and Table 1 summarize the data on electrical conductivity and tensile strength in various works and the current work. It can be concluded that there are few reports on Al/Gr composites with improved electrical conductivity compared to that of pure aluminum. Most researchers focus on improving mechanical properties, but the corresponding electrical conductivity of the material decreases. Here, this work shows an electrical conductivity of 66.63% IACS, which reflects a great improvement in the Al/Gr composites, and the tensile strength is also increased by about 11% compared that of pure Al bulk.

The improvement in the electrical conductivity of Al is very important and useful for practical applications. It is well known that there is a trend to use Al instead of Cu for electric transport due to the lower weight and cost of Al-based materials. Very recently, Al has been used in electrical vehicles as the wire harness, and it might also be widely used in traditional electric cables. The improvement achieved in this work would greatly reduce the energy losses and also the heat production during the power transmission process for reaching an efficient use of energy. Therefore, it is very significant for scaling up the production of this layer-structured Gr/Al composite. Differing from the method in this work, the large-scale production would be realized based on a roll-to-roll fabrication procedure. A specially designed machine would be built to combine the fabrication process in this work in a roll-to-roll way, which would be studied in near future.

## 4. Conclusions

In summary, a novel layer-structured Al/Gr composite was successfully designed and prepared with the help of ultrasonication spraying of graphene on Al foil and an acid treatment to remove native alumina. The layer-structured Al/Gr composite with graphene-coated Al foil as building blocks presents an enhancement in both electrical conductivity and engineering strength, with good interconnection between different layers. In particular, the electrical conductivity of Al/Gr50 researches a highest value of 66.63% IACS, which is 11% higher than that of a pure Al matrix. Considering that the electrical performance of Al-based alloy usually decreases dramatically, the improvement achieved in the layer-structured Al/Gr composite in this work would provide a new way to fabricate Al alloys with new performance, which is very significant and important for practical applications.

## Figures and Tables

**Table 1:** The detailed preparation process and specific properties of Al/Gr composites shown in Figure 5.

Icon	Composites	Preparation Method	Tensile Strength MPa	Electrical Conductivity %IACS	Ref.
★	Gr/Al	Ultrasonic spraying Gr + VHP	96.39	66.63	This work
●	Gr/Al	Casting + cold rolling + aging treatment	180	63.45	[28]
▼	Gr/Al8030	Powder modification + semi-solid extrusion	213	61.86	[40]
⬟	FLG/Al	Solution mechanical stirring + SPS + hot extrusion	165	59.8	[38]
◄	FLG/Al	Colloidal mixing + SPS	173	59.13	[41]
►	Gr/1060Al	FSP + hot extrusion	149	58.8	[42]
●	GO/Al	Ultrasonic mixing in colloids + SPS	176	58.6	[43]
⬢	GNPs/Al	Mechanical stirring in ethanol + high temperature sintering	250	56	[44]
◆	Gr/Al	Mechanical ball milling + hot extrusion	131	52	[36]
▼	GNS/Al6063	Mechanical ball milling + hot extrusion	276	52.9	[24]
►	rGO/Al	Solution process mixing + thermal reduction	374	51	[27]
⬟	GNS/Al6061	Mechanical ball milling + hot extrusion	256	40.6	[45]

## Data Availability

All data that support the findings of this study are included within the article (and any supplementary files).

## Supplementary Materials

The following supporting information can be downloaded at: https://www.mdpi.com/article/10.3390/ma17122979/s1, Figure S1: OM images of Al/Gr composite foils. (a) Al/Gr10. (b) Al/Gr20. (c) Al/Gr50 (d) Al/Gr80; Figure S2. SEM images of the cross section of Al/Gr composites. (a) Unpickled pretreatment. (b) Pickled pretreatment; Figure S3. Measuring principles diagram of the van der Pauw method; Figure S4. The thickness of Al/Gr composites with different graphene spraying times; Figure S5. The electrical conductivities of Al/Gr50 composites with hot pressing at different temperatures and at the same pressure of 50 MPa; Figure S6. (a,b) Tensile sample diagram. SEM morphology of the fracture surface of bulk Al/Gr bulk composites. (c) Pickled Al/Gr50. (d) Unpickled Al/Gr50. (e) Pickled Al/Gr80. (f) Unpickled Al/Gr80.
