# Supplementary material for: Building a Layer-Structured Aluminum/Graphene Composite with Significant Improvement in Electrical Conductivity
Source: Materials (Basel). 2024 Jun 18;17(12):2979. doi: 10.3390/ma17122979 (PMC11205440; doi:10.3390/ma17122979)
Supplement: Supplementary file 1 [file materials-17-02979-s001.zip › materials-3019092-supplementary.pdf]

Supporting Information

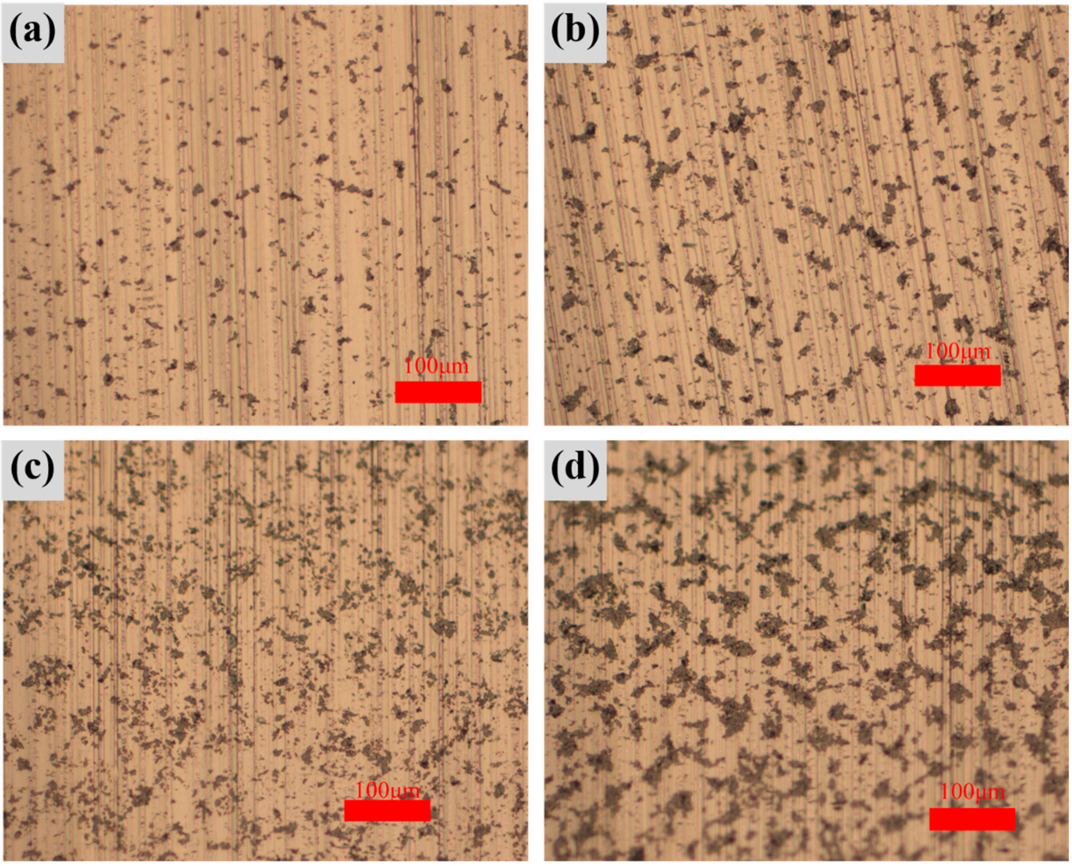

Figure S1. OM images of Al/Gr composite foils. (a)Al/Gr10. (b)Al/Gr20. (c) Al/Gr50 (d) Al/Gr80.

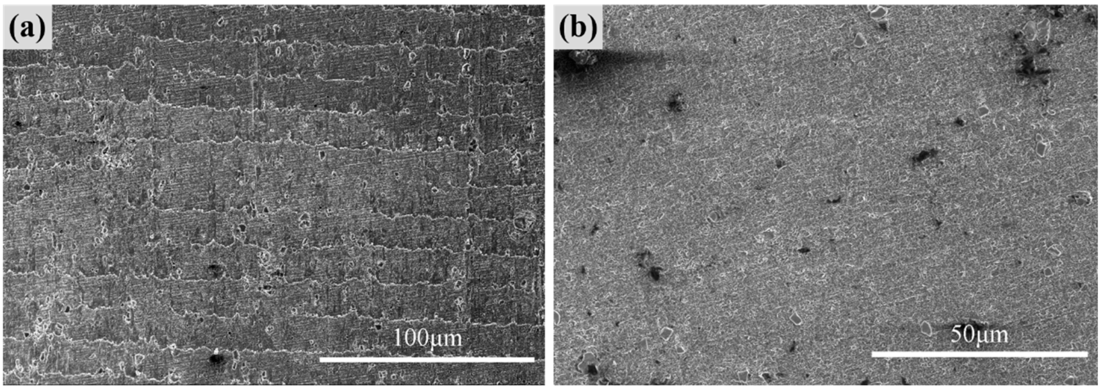

Figure S2. SEM images of cross-section of Al/Gr composites. (a) unpickled pretreatment. (b) pickled pretreatment.

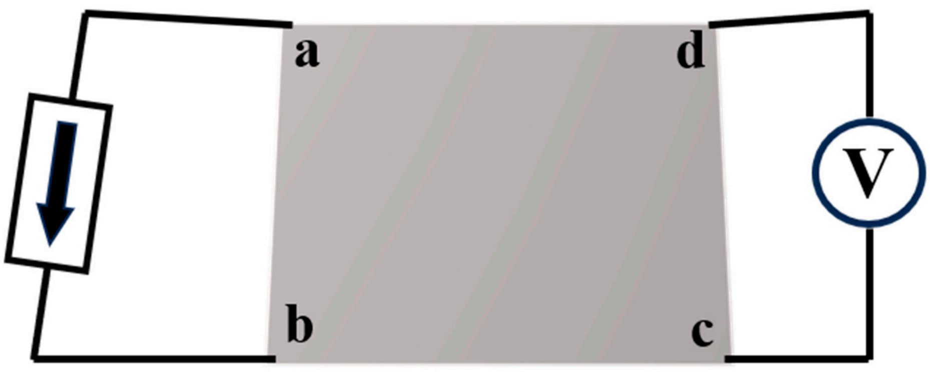

Figure S3. Measuring principles diagram van der Pauw method. The electrical conductivity measurement was carried out using four-probe staion after the sample surface were carefully polished. Before the actual measurement, metal probes a, b, c and d were arranged around the corners of the sample. When a certain current I appears between probe a and probe b, a corresponding potential difference V will be generated between the probe d and probe c. The materials resistance is then calculated according to the equation (1):

$$\mathrm{R} = \frac{U}{I}$$

Subsequently, multiple measurements were conducted around the sample to obtain R_{ab}, R_{ac}. Because the probe position is not completely square and symmetrical, a correction factor for the probe position is required for calculation.

$$\mathrm{f} = 1 - \left(\frac{R_{ab} - R_{ac}}{R_{ab} + R_{ac}}\right)^2 * \frac{ln2}{2} - \left(\frac{R_{ab} - R_{ac}}{R_{ab} + R_{ac}}\right)^4 * \left(\frac{(ln2)^2}{2} - \frac{(ln2)^3}{2}\right)$$

The resistivity value can then be obtained by the following equation (3),

$$\rho = \frac{\pi \hbar}{2ln2}(R_{ab} + R_{ac}) * f$$

where $\hbar$ is the average thickness of material. The thickness of Al/Gr bulk composite is calculated by measuring density, weight and area. Finally, the electrical conductivity can be calculated as the reciprocal of the resistivity. It should be noted that due to the difference of sample measurement temperature, it also needs to be corrected.

$$\sigma_{20\,℃} = \frac{1 + 0.00393 * (t - 20)}{\rho}$$

$$\mathrm{C} = \sigma_{IACS} = \frac{\sigma_{20\,℃}}{58000000}$$

In order to ensure the accuracy of the measurement，The samples were polished by using particles in order to avoid any rough-surface-effects affecting the accuracy of the conductivity measurement.

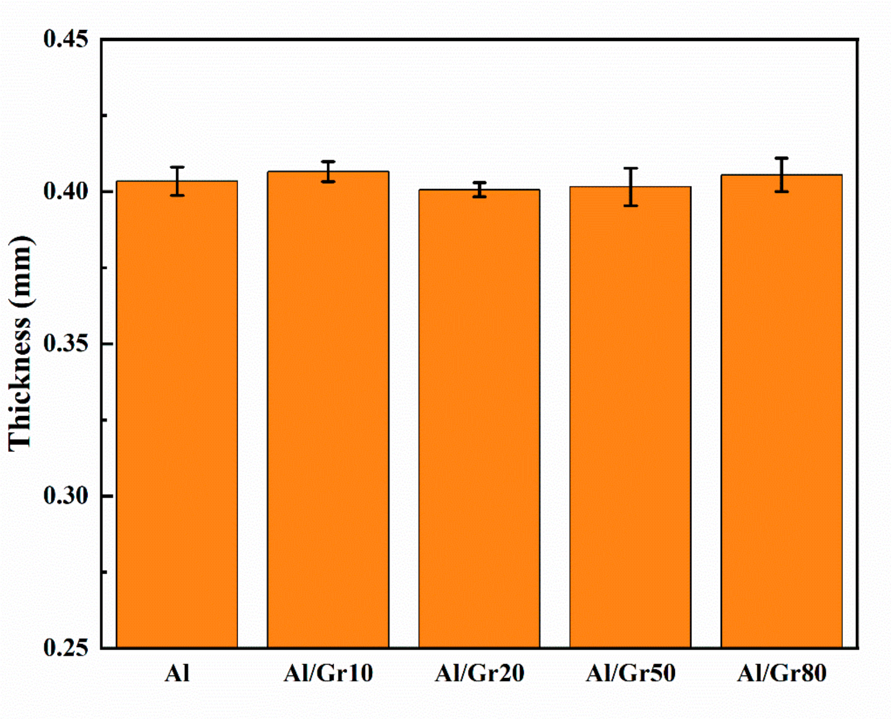

Figure. S4 The thickness of Al/Gr composites with different graphene spraying times.

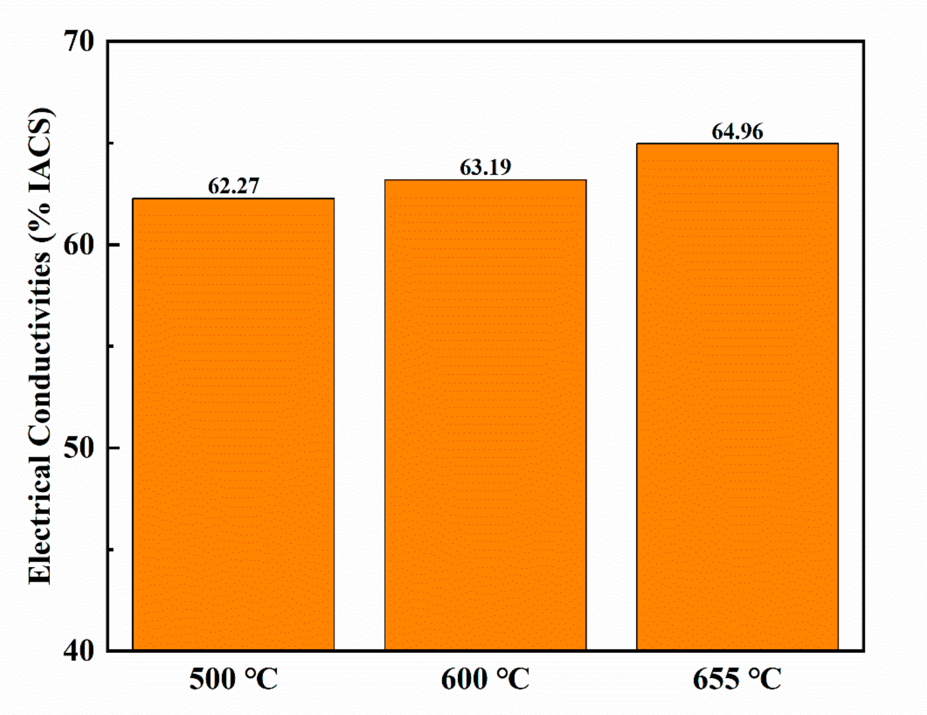

Figure.S5 The electrical conductivities of Al/Gr50 composites with hot pressing at different temperatures and at the same pressure of 50 MPa

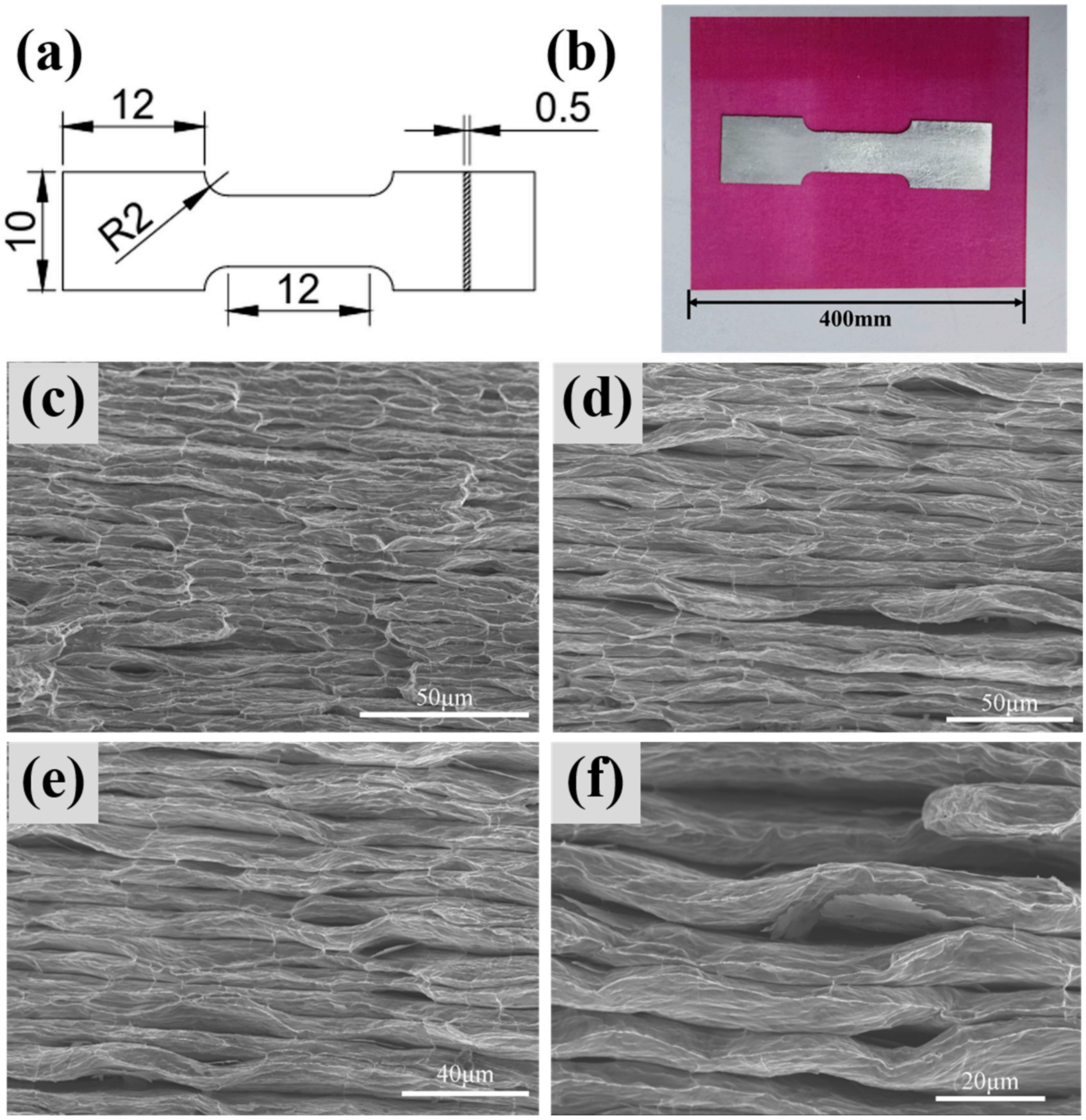

Figure S6. (a-b) Tensile Sample Diagram. SEM morphology of the fracture surface of bulk Al/Gr bulk composites. (c) Pickled Al/Gr50. (d) Unpickled Al/Gr50. (e) Pickled Al/Gr80. (f) Unpickled Al/Gr80.
